# Capturing and genotyping the genome-wide genetic diversity of trees for association mapping and genomic selection

**DOI:** 10.1186/1753-6561-5-S7-I7

**Published:** 2011-09-13

**Authors:** Matias Kirst, Márcio Resende, Patricio Munoz, Leandro Neves

**Affiliations:** 1 School of Forest Resources and Conservation, University of Florida, P.O. Box 110410, Gainesville, FL 32611, USA; 2 Genetics and Genomics Graduate Program, University of Florida, P.O. Box 110690, Gainesville, FL 32611, USA; 3 Plant Molecular and Cellular Biology Graduate Program, University of Florida, P.O. Box 110690, Gainesville, FL 32611, USA

## Background

Growing demand for food and fiber, and a rapidly changing climate will require that plant breeders accelerate the improvement of germplasm adapted to new sources of biotic and abiotic stress. In trees, the threat from climate change is more evident and the solutions more challenging than in any other plant species, due to the complexity and cost of breeding programs, and the long breeding cycles. Therefore, the discovery of genetic polymorphism that can be exploited for early selection of better adapted and productive individuals is essential. Quantitative trait loci (QTL) analysis provided an initial glimpse at the architecture of complex traits, but limited transferability across populations and resolution hampered the adoption of markers in tree breeding programs. Recently, association studies have become the method of choice for detection of markers implicated in trait variation, because of higher resolution, population transferability and allelic diversity captured relative to the QTL approach. However, in tree species, association studies have been largely constrained to sampling the genetic diversity in a limited fraction of the genome, and in small populations. Evidence from genome-wide association studies (GWAS) in humans and advanced crops clearly show that larger populations, and the sampling of regulatory variants and rare alleles is critical to dissect the genetic control of complex traits for marker-assisted breeding (MAB). As the limitations of QTL and GWAS approaches become evident, "hybrid" intermediate strategies that combine the advantages of both methods have emerged. Notably, genomic selection has become an alternative to MAB. Genomic selection (GS), which relies on developing genome-wide marker-based models that predict the genetic value of progeny, will be particularly valuable for early selection in tree breeding programs. However, the implications of GS may also be highly valuable to identify mating designs that generate progeny with optimal allelic combinations for superior growth and wood properties, and adaptive capabilities.

## Methods

To address limitation of recent association studies in forest tree species we have adopted an approach that combines targeted sequence-capture followed by high-throughput sequencing, to genotype eastern cottonwood (*Populus deltoides*) and loblolly pine (*Pinus taeda*) populations. To identify genetic polymorphisms that regulate biomass productivity, wood quality, and disease resistance, we have optimized methods of sequence-capture targeted regions of the genome for unbiased, high-throughput and low cost recovery of coding and regulatory sequences. This establishes the foundation for GWAS in both species, addressing a critical limitation of these studies in tree genomes – i.e. sampling of regulatory variants and rare alleles.

While the work described above resolves part of the challenges of association studies, the use of GWAS will be most useful for discovery of genes to be targeted for genetic modification, rather than MAB. This is the case because the fraction of the total genetic variation explained by association studies is likely to remain small, considering the limitations of the existing populations. In an effort to implement marker-based breeding, we recently completed the first assessment of the utility of GS in an experimental population of loblolly pine, where we explored the contribution of factors such as age of model estimation and site location on the accuracy of prediction models. Furthermore, the incorporation of non-additive effects to prediction models has been evaluated for improvement of their accuracy.

## Results and conclusions

Two association studies are currently underway, in loblolly pine and *Populus deltoides*, where a large fraction of the coding and regulatory sequences of their genomes are being re-sequenced for SNP detection. Existing results from sequence capture indicate that the majority of targeted regions can be effectively captured, even in genomes of very high complexity, such as that of loblolly pine. In parallel we have now verified the suitability of applying genomic selection to a breeding population of loblolly pine. Estimates generated for prediction models developed for this population indicates that accuracies that are comparable to traditional phenotypic selection can be obtained. Therefore, considering the significant reduction in the breeding cycle length due to early identification of elite genotypes, the increase in efficiency per unit of time in the selection response of genomic selection is almost twice as high, compared to traditional breeding. By combining GS with advanced methods of vegetative propagation, breeding and seedling production, a breeding cycle can be reduced from decades to less than 5 years. While early selection of superior genotypes is an obvious application of GS to tree improvement programs, prediction models can be utilized to guide the mating design of future breeding cycles to favor stacking of favorable alleles over multiple generations. Towards this end, we have initiated crosses aimed at generating families that are predicted to have exceptional adaption, as well as biomass growth and quality properties for bioenergy, pulp and paper and timber production.

